# Smoking, smoking cessation, and 7-year mortality in a cohort of Thai adults

**DOI:** 10.1186/s12963-015-0062-0

**Published:** 2015-10-27

**Authors:** Jiaying Zhao, Cha-aim Pachanee, Vasoontara Yiengprugsawan, Sam-ang Seubsman, Adrian Sleigh, Thai Cohort Study Team

**Affiliations:** National Centre for Epidemiology and Population Health and Global Health Division, Research School of Population Health, The Australian National University, Canberra, Australia; International Health and Policy Program, Bureau of Policy and Strategy, Ministry of Public Health, Nonthaburi, Thailand; School of Human Ecology, Sukhothai Thammathirat Open University, Nonthaburi, Thailand

**Keywords:** Smoking, Tobacco, Cessation, Mortality, Cardiovascular diseases, Thailand

## Abstract

**Background:**

Smoking is a strong risk factor for mortality in both the developed and the developing world. However, there is still limited research to examine the impact of smoking cessation and mortality in middle-income Southeast Asian populations.

**Methods:**

We use longitudinal data from a large Thai cohort of adult Open University students residing nationwide, linked with official death records to assess the association of smoking status and mortality risks during a 7-year follow-up. The log-rank test was used to evaluate the statistical probability of differential survival according to baseline smoking status. Multivariate hazard ratios (HR) were reported for smoking status and all-cause and cause-specific mortality.

**Results:**

From 2005 baseline to 2012, current smokers were more likely to die than cohort members who ceased smoking and never smokers (1.9 vs 1.3 vs 0.6 %, *p* < 0.05). The hazard of all-cause mortality increased with the daily amount of cigarette consumption among both current and former smokers. Cause of death analyses showed that current male smokers had a significantly increased risk of cardiovascular disease related mortality (HR 3.9 [95 % CI 1.8–8.1]). Former male smokers had a moderate increase in risk of dying from cardiovascular diseases compared to never smokers (HR 1.6 [95 % CI 0.7–3.4]). Current male smokers between 2005 and 2009 experienced highest subsequent mortality hazards during the period 2009–2012 compared to never smokers (HR 2.1 [95 % CI 1.4–3.4]). The higher risk of dying reduced if people quit smoking during the 2005–2009 follow-up period (HR 1.5 [95 % CI 0.7–3.3]). Risk for mortality fell even further among long-term quitters (HR 1.4 [95 % CI 0.9–2.2]).

**Conclusion:**

Among a large nationwide cohort of Thai adults, current smokers were at a significantly and substantially higher risk of all-cause mortality, especially cardiovascular-related mortality. The higher risk of dying fell if people quit smoking and the risk for mortality was even lower among long-term quitters. Promotion of smoking cessation will contribute substantially to the reduction in avoidable mortality in Thailand.

## Introduction

Smoking is a prominent risk factor of mortality in both the developed and the developing world [1–3]. In 2010, tobacco smoking was the second-leading risk factor for global burden of diseases [4, 5]. Notably, a 50-year prospective study of British doctors documented a secular improvement in longevity for never smokers, but not for men who continued smoking cigarettes [6]. Moreover, among smokers, heavy smokers had higher mortality risk [6, 7]. Similar results were found from 20-year follow-up in the Nurses’ Health Study [8].

Previous literature has shown that smoking cessation can reduce overall mortality risk [9–12] and smoking cessation can also lower mortality from specific causes such as cancer [13, 14], respiratory diseases [15, 16], cardiovascular disease [17–19], and injury [20, 21]. However, due to racial, cultural, and smoking behavior differences (e.g.*,* age of initiation, duration), smoking and cessation patterns may differ across populations. Therefore, local mortality data in relation to smoking cessation are essential to understand potential population benefits for public health campaigns to promote smoking reduction.

Mortality risks decrease with the length of time since quitting smoking; in the UK, for example, it was estimated that quitting smoking at age 60, 50, 40, or 30 gained about 3, 6, 9, or 10 years of life expectancy, respectively [7]. A large population-based Japanese cohort study underway since 1950 showed that for heavy smokers, life expectancy was reduced by almost a decade (8 years for men and 10 years for women) [22]. To date, in Asia, most studies of smoking cessation and mortality have been among more affluent East Asian countries [12, 13, 17, 19]. There is limited research to date examining how quickly the benefits of smoking cessation can be observed after quitting smoking in middle-income Southeast Asian populations.

Thailand, a developing country in Southeast Asia, has undergone rapid economic growth and a health-risk transition in recent decades, moving from malnutrition and infectious diseases to chronic diseases as major causes of death. In 2009, smoking contributed to the highest death rate and was also the second-leadingrisk factor contributing to burden of disease among Thai males [23]. Smoking-related deaths (lung cancer, heart disease, chronic obstructive pulmonary disease) increased from 45,136 cases in 2004 to 50,710 cases in 2009 [23]. The overall smoking rate in Thailand had decreased from 32.0 % in 1991 to 19.9 % in 2013 and 20.7 % in 2014 [24]. A recent Global Adult Tobacco Survey suggested that Thailand experienced a significant decline in the percentage of smokers who made a quit attempt during the past 12 months from 2009 (49.8 %) to 2011 (36.7 %) [25]. Almost all adults (>97.0 %) believed that smoking caused serious illness and lung cancer but fewer (around 80 %) thought smoking caused stroke and heart attack. This may be partly because local evidence of smoking related to specific diseases and benefits of quitting smoking is still rare.

We use data from a large Thai cohort made up of adult Open University students residing nationwide, linked with official death records to assess the association with reported smoking status and mortality risks within seven year follow-up. We present the relationship between smoking status, including average number of cigarettes smoked daily, and subsequent all-cause and cause-specific mortality. We compare mortality risk among never smokers, long-term quitters (quit more than 4 years), new quitters (quit within 4 years), and current smokers. Our findings provide empirical evidence on smoking cessation and mortality in middle-income Southeast Asia.

## Methods

### Study population

We use the Thai Cohort Study (TCS) data, which collected information on health status as well as risk and protective factors associated with socioeconomic development. The study included 87,151 adult distance-learning students enrolled at Sukhothai Thammathirat Open University, residing all over Thailand in 2005. The participants represented the Thai population well in terms of social geography, religion, socioeconomic status, and income [26, 27]. TCS participants were followed up in 2009 by mail and 60,569 participants completed questionnaires for an overall response rate of 71 % [28].

TCS data were linked to official mortality data from the Thai Ministry of Interior using the 13-digit citizen identification number, a unique ID for each Thai person. Up until August 15, 2012, 767 deaths were recorded among the cohort members. The coverage of registration of adult deaths in Thailand was 86 % from 1950 to 2000 [29], which improved further to 95 % over the period reported in this study [30].

Cause-of-death information occurring before the end of 2010 (*n* = 583) was provided and verified by the Ministry of Public Health. Causes of deaths which were ill-defined had been investigated by the vital statistics office of the Ministry of Public Health according to hospital records and verbal autopsies. Among our analyses, ill-defined deaths were only 8.4 % (*n* = 49) of the total number of deaths in the cohort.

### Measurements

Smoking status in 2005 and 2009 was categorized into never smoker, former smoker, or current smoker. In 2005, both current and former smokers were asked to note average number of cigarettes smoked daily. We further categorized smoking status in 2005 into never smoker, former light smokers (number of cigarettes <10), former medium smokers (number of cigarettes 10–19), former heavy smokers (number of cigarettes > =20), current light smokers (number of cigarettes <10), current medium smokers (number of cigarettes 10–19), current heavy smokers (number of cigarettes > =20). For 4-year smoking status, we combined dynamic smoking status from 2005 to 2009 into four categories: never smoker (2005–09), those who had stopped smoking from 2005, those who had stopped smoking in 2009, and current smoker in 2005 and 2009.

Analyses included baseline data on sex, birth year, urban or rural residence, income, and health insurance coverage. Personal monthly income measured in 2005 was classified as ≤3000, 3001–20,000, or >20,000 Baht (1 $US ~ 30 Baht). Household monthly income measured in 2009 was classified as <7000, 7001–30,000, or >30,000 Baht. Health insurance is provided through three programs: the civil servant scheme, the Universal Coverage scheme available to Thai nationals (initially called the 30 Baht scheme), and others including social security for private employees.

Potential confounders related to health behaviors including alcohol drinking and physical activity in 2005 and 2009 were also analysed. We classified alcohol consumption in 2005 as occasional social drinker, never drinker, current regular drinker, or now stopped. In 2009, the categories included non-drinker, light drinker (≤7 glasses per week), or moderate or heavy drinker (≥8 glasses per week). We recoded weekly physical activity in 2005 and 2009 as less than 7 sessions or 7 sessions or more, based on standard measures from the International Physical Activity Questionnaire and Active Australia Survey [31].

Self-rated health was used to as an indicator of overall general health status at baseline, assessed with a standardized question, “Overall, how would you rate your health over the last 4 weeks?” in both 2005 and 2009. Responses were categorized as excellent, very good, good, fair, poor, and very poor. We divided self-rated health into positive (excellent/very good/good/fair) and negative (poor and very poor) groups. Body mass index (BMI) in 2005 and 2009 was based on self-reported height and weight. We used Asian BMI standards: underweight (<18.5), normal (18.5 to <23), overweight at risk (23 to <25), obese I (25 to <30), and obese II (≥30) [32]. In a separate study, self-reported height and weight was validated and showed the estimates were reasonably accurate and suitable for use in a large cohort study [33].

### Statistical analyses

We examined the distribution of cohort smoking status in 2005 by potential confounders (e.g.*,* main socio-demographic attributes, risk behaviors, and physical activity). We examined the distribution of smoking by survival status on December 31, 2010 and August 15, 2012. For males we also assessed the distribution of smoking status, including the information on the daily average number of cigarettes smoked in 2005 by survival status. Kaplan Meier survival curves display differential survival patterns by baseline self-rated health (March 1, 2005). End-point events were all-cause deaths and cause-specific deaths until December 31, 2010 (cardiovascular diseases, cancers, injury, and other causes) and all-cause deaths until August 15, 2012. We used the log-rank test to test the statistical probability of observed difference in survival patterns according to 2005 smoking status.

As males constituted the vast majority of smokers in the cohort [34], multivariate regression analyses only focused on males. Multivariate hazard ratios (HR) and 95 % confidence intervals for mortality by baseline (2005) smoking status were estimated using the Cox model after confirming that the assumption of proportionality of hazards was held. For each of four specific death outcomes (cardiovascular diseases, cancers, injury, and other causes), models were developed in the same way as for all-cause mortality.

We performed separate Cox regression analyses examining all-cause mortality in the cohort between 2009 and 2012 in relation to longitudinal dynamic smoking status (2005–2009). We used ‘never smoker’ as a reference. The end-point events for those who were followed up in 2009 were all-cause deaths until August 15, 2012. Covariates include birth year and health insurance coverage in 2005, as well as other four-year follow-up information (2009) including residence, monthly household income, drinking, physical activity, body mass index, and self-reported overall health.

### Ethical approval

Informed written consent was obtained from all participants. All students were advised that they could withdraw, or not participate, without any effect on their academic progress. The questionnaires never sought sensitive personal information and no biological samples were taken. Ethics approval was obtained from Sukhothai Thammathirat Open University Research and Development Institute (protocol 0522/10) and the Australian National University Human Research Ethics Committee (protocols 2004/344 and 2009/570).

## Results

### Distribution of smoking by potential confounders

Smoking experience was reported frequently among males and infrequently among females (Table 1). The proportion of females who never smoke was more than 90 % in the cohort.

**Table 1:** Socio-demographic attributes at baseline (2005) by smoking status and sex in the Thai Cohort Study

Socio-demographic attributes		Column % distribution of smoking status by sex								
		Total (*n* = 84,573)			Males (*n* = 38,450)			Females (*n* = 46,123)		
		Never (*n* = 61,143)	Former (*n* = 14,698)	Current (*n* = 8732)	Never (*n* = 17,653)	Former (*n* = 12,542)	Current (*n* = 8255)	Never (*n* = 43,490)	Former (*n* = 2156)	Current (*n* = 477)
Birth Year	−1959 (> = 46 years)	4.3	12.3	7.8	6.2	13.9	8.1	3.6	3.0	2.9
	1960–1969 (36–45 years)	18.3	28.4	24.5	20.4	30.2	24.7	17.4	17.9	21.4
	1970–1974 (31–35 years)	16.3	20.0	22.3	17.4	20.1	22.5	15.8	19.6	19.5
	1975–1979 (26–30 years)	25.7	22.6	27.0	25.3	21.2	26.8	25.9	30.6	31.7
	1980–(<=25 years)	35.4	16.7	18.3	30.7	14.6	18.0	37.4	28.9	24.5
Personal monthly income (Baht)	<3000	11.4	8.7	12.4	13.1	8.7	12.6	10.6	8.2	8.4
	3001–20,000	79.3	75.7	76.3	74.5	74.6	76.4	81.3	81.9	75.6
	> = 20.001	9.3	15.7	11.3	12.4	16.7	11.0	8.1	9.8	16.1
Place of residence	Urban	51.4	53.0	52.0	49.4	51.3	50.9	52.2	63.2	71.2
	Rural	48.6	47.0	48.0	50.6	48.7	49.1	47.8	36.8	28.8
Health Insurance Coverage	30 Baht scheme	13.7	11.4	13.0	13.4	11.1	13.2	13.8	13.0	8.4
	Civil servant scheme	23.2	31.4	27.2	29.0	34.1	28.0	20.8	15.8	12.4
	Others	63.2	57.2	59.9	57.6	54.8	58.7	65.4	71.2	79.2
Alcohol consumption	Occasional	56.5	67.8	66.7	69.5	67.3	66.1	51.2	70.9	76.1
	Never	35.6	3.2	3.3	19.7	2.8	3.3	42.1	5.8	3.6
	Regular	1.3	11.5	18.6	3.7	12.9	18.8	0.4	3.7	14.0
	Stopped	6.6	17.4	11.4	7.1	17.1	11.7	6.3	19.6	6.3
Weekly physical activity	<7 sessions	35.9	29.2	27.3	24.4	26.5	26.1	40.6	45.0	49.4
	7+ sessions	64.1	70.8	72.7	75.6	73.5	73.9	59.4	55.0	50.6
Body mass index	Underweight(<18.5)	17.9	6.2	6.9	7.6	4.1	5.9	22.1	18.4	22.7
	Normal (18.5 to <23)	57.0	46.4	49.2	53.1	44.6	49.0	58.6	56.7	52.2
	Overweight at risk (23 to <25)	12.7	22.1	20.4	20.3	24.1	20.8	9.6	10.4	12.1
	Obesity I (25 to <30)	10.1	21.9	20.3	16.2	23.8	21.0	7.6	11.0	8.5
	Obesity II (≥30)	2.3	3.4	3.3	2.8	3.4	3.2	2.1	3.6	4.5
Poor self-reported health	No	95.5	95.0	95.4	96.9	95.6	95.6	95.0	91.4	91.4
	Yes	4.5	5.0	4.6	3.1	4.4	4.4	5.0	8.6	8.6

Among males, more than 90 % of cohort members were born after 1960 (aged ≤45 years in 2005 baseline). Never smokers had a higher proportion of younger birth cohort (born 1980 or after, aged ≤25 years in 2005 baseline). The proportion of male current smokers were similar across birth cohorts – except for low proportions among the oldest (born before 1960) and the youngest (born in 1980 or after). Lower-income males were more likely to be current smokers, and less likely to quit smoking. The difference in smoking status between rural and urban males was modest. Males who reported being regular alcohol drinkers were more likely to be current smokers. Compared with never smokers, current smokers and former smokers had higher proportions reporting poor health.

### Smoking and mortality

From baseline (2005) to 2012, current smokers were more likely to die than people who quit smoking and those who never smoked (1.9 vs 1.3 vs 0.6, *p* < 0.05) (Table 2). The patterns of association between smoking status and mortality risk for males were similar to those for the whole cohort. For males, former heavy smokers (2.5 %) were more likely to die compared with former medium smokers (1.7 %) and former light smokers (0.9 %). A similar trend for heavy, medium and light smokers was observed among current smokers (2.7 vs 2.2 vs 1.5 %, *p* < 0.05).

**Table 2:** Survival data by smoking status (row percent) for the Thai Cohort Study, 2005–2012

		End-point follow up (row %)							
Sex	Smoking status (2005)	August 15, 2012		December 31, 2010					
		Survival status		Survival status		Deaths by causes			
		Survived	Died	Survived	Died	Cardiovascular	Cancer	Injury	Other
Total	Never	60,758	394 (0.6 %)	60,848	304 (0.5 %)	31 (0.1 %)	68 (0.1 %)	115 (0.2 %)	90 (0.1 %)
	Former	14,513	189 (1.3 %)	14,563	139 (0.9 %)	20 (0.1 %)	32 (0.2 %)	35 (0.2 %)	52 (0.4 %)
	Current	8569	163 (1.9 %)	8608	124 (1.4 %)	25 (0.3 %)	16 (0.2 %)	49 (0.6 %)	34 (0.4 %)
	Total	83,840	746 (0.9 %)	84,019	567 (0.7 %)	78 (0.1 %)	118 (0.1 %)	204 (0.2 %)	183 (0.2 %)
Males	Never	17,477	179 (1.0 %)	17,517	139 (0.8 %)	16 (0.1 %)	23 (0.1 %)	63 (0.4 %)	37 (0.2 %)
	Former All	12,369	174 (1.4 %)	12,414	129 (1.0 %)	19 (0.2 %)	28 (0.2 %)	33 (0.3 %)	49 (0.4 %)
	Former light smokers	7011	64 (0.9 %)	7026	49 (0.7 %)	7 (0.1 %)	7 (0.1 %)	16 (0.2 %)	19 (0.3 %)
	Former medium smokers	3197	54 (1.7 %)	3211	40 (1.2 %)	5 (0.2 %)	10 (0.3 %)	8 (0.3 %)	17 (0.5 %)
	Former heavy smokers	2161	56 (2.5 %)	2177	40 (1.8 %)	7 (0.3 %)	11 (0.5 %)	9 (0.4 %)	13 (0.6 %)
	Current All	8098	157 (1.9 %)	8136	119 (1.4 %)	24 (0.3 %)	14 (0.2 %)	49 (0.6 %)	32 (0.4 %)
	Current light smokers	3886	57 (1.5 %)	3902	41 (1.0 %)	6 (0.2 %)	4 (0.1 %)	21 (0.5 %)	10 (0.3 %)
	Current medium smokers	3035	67 (2.2 %)	3048	54 (1.7 %)	12 (0.4 %)	8 (0.3 %)	18 (0.6 %)	16 (0.5 %)
	Current heavy smokers	1177	33 (2.7 %)	1186	24 (2.0 %)	6 (0.5 %)	2 (0.2 %)	10 (0.8 %)	6 (0.5 %)
	Total	37,944	510 (1.3 %)	38,067	387 (1.0 %)	61 (0.2 %)	65 (0.2 %)	150 (0.4 %)	123 (0.3 %)

There were 78 cohort deaths from cardiovascular diseases, 118 deaths from cancers, 204 from injury, and 183 deaths from other causes of death from baseline in 2005 up to 2010. Cause-specific analyses suggested that current smokers for both total population and males were more likely to die from cardiovascular diseases (CVD), injury, and from other causes (*p* < 0.05) than those who had never smoked. In addition, current smokers among the whole cohort and particularly males also had a higher risk of dying from cardiovascular diseases and injury than former smokers (*p* < 0.05). However, the risk of dying from cancers across different groups by smoking status was not significant.

**Fig. 1:**
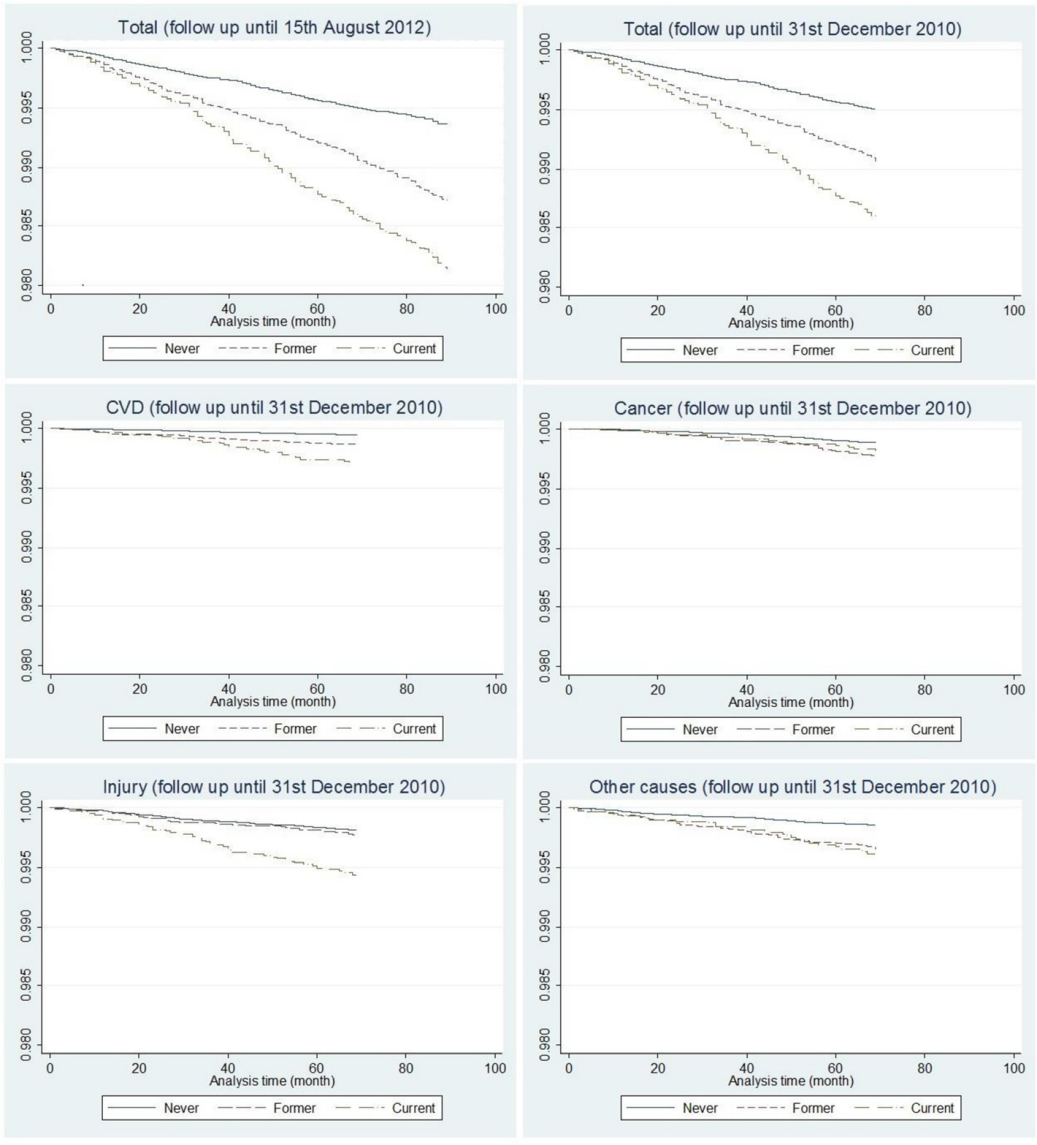
Kaplan-Meier survival curves for smoking status (2005) by cause of death. Note: y axis records the proportion surviving; x axis records the duration of survival in month

**Fig. 2:**
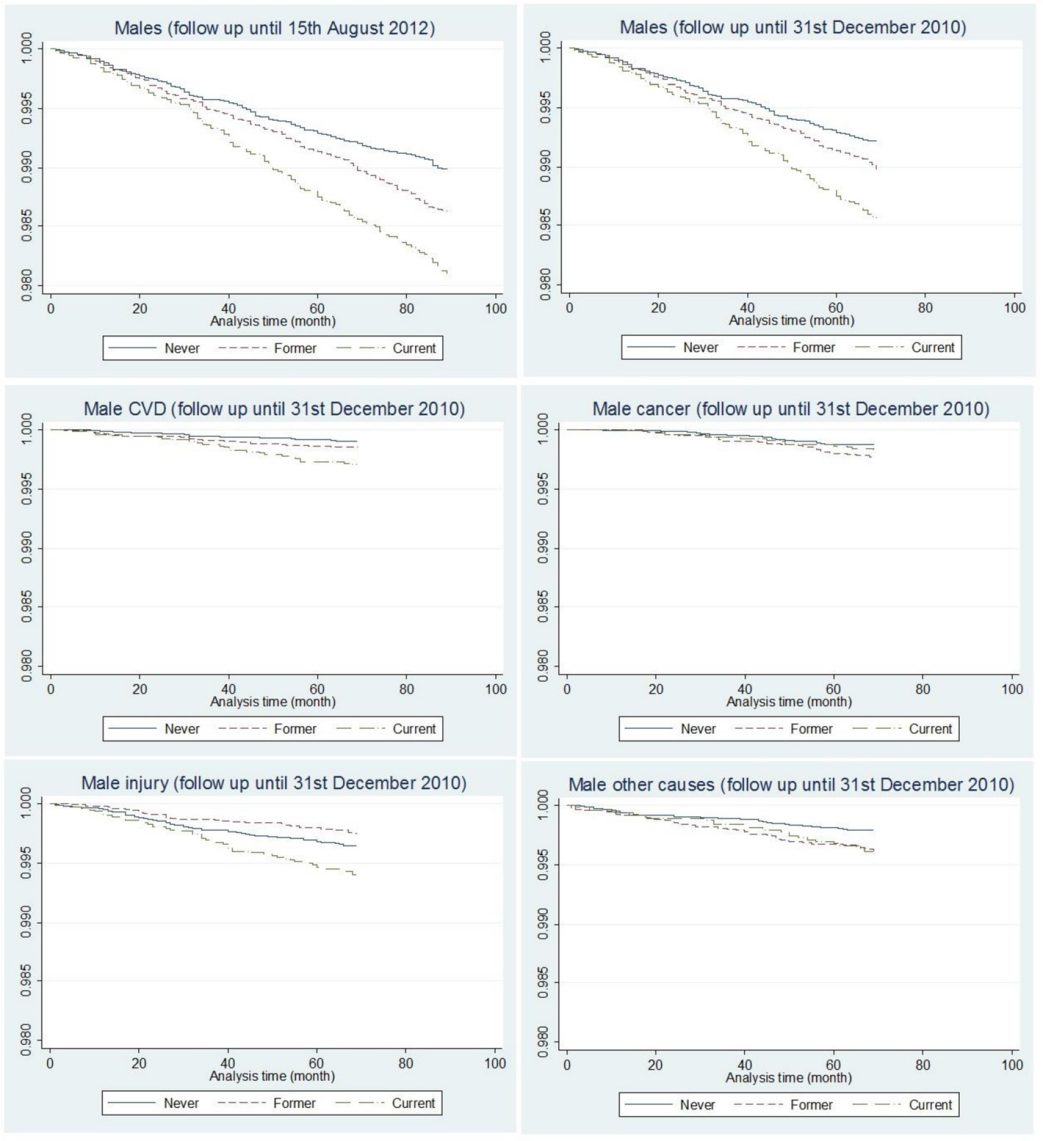
Kaplan-Meier survival curves for smoking status (2005) by cause of death, males. Note: y axis records the proportion surviving; x axis records the duration of survival in month

Kaplan-Meier survival curves by smoking status (Fig. 1) indicated that current smokers had a higher risk of all-cause and cause-specific deaths (i.e., cardiovascular diseases, cancer, injury, and other causes of death) than never smokers (log-rank test *p* < 0.05). Cohort members who stopped smoking also reduced the risk of mortality from all-cause mortality, cardiovascular diseases, and injury compared with current smokers. However, mortality from cancers and other causes of death did not significantly differ between current smokers and former smokers. The survival patterns for males were similar to those among the whole cohort (Fig. 2).

As there were very few female deaths among current smokers (*n* = 6) and former smokers (*n* = 15), the analyses of the Cox hazard model are only conducted for males. In the Cox proportional hazard model, current smokers at baseline, after controlling for age, rural or urban residence in 2005, monthly income, alcohol drinking, physical activity, body mass index, and self-reported health had a significantly higher risk of subsequent death till 2012 (HR 1.8 [95 % CI:1.4–2.3]) than never smokers (Table 3). Former smokers also had a higher risk of mortality than never smokers, but this effect was not significant. The hazard ratio of premature mortality for former smokers was much lower than that of current smokers (HR 1.2 [95 % CI:0.9–1.5]).

**Table 3:** Mortality and smoking status (2005) for males in the Thai Cohort Study, 2005–2012

Smoking status (2005)	All-causes (to Aug 2012)	All causes (till Dec 2010)	Causes of death			
			Cardiovascular	Cancer	Injury	Other
Former	1.2 [0.9–1.5]	1.1 [0.9–1.5]	1.6 [0.7–3.4]	1.0 [0.5–1.8]	0.8 [0.5–1.3]	1.6 [1.0–2.6]
Current	1.8 [1.4–2.3]	1.8 [1.3–2.3]	3.9 [1.8–8.1]	0.9 [0.4–2.0]	1.7 [1.1–2.6]	1.8 [1.1–3.0]
Former light smokers	0.9 [0.7–1.2]	--	--	--	--	--
Former medium smokers	1.5 [1.0–2.1]	--	--	--	--	--
Former heavy smokers	1.7 [1.2–2.5]	--	--	--	--	--
Current light smokers	1.6 [1.2–2.2]	--	--	--	--	--
Current medium smokers	1.9 [1.4–2.7]	--	--	--	--	--
Current heavy smokers	2.4 [1.6–3.6]	--	--	--	--	--

Hazard ratios compare mortality over the follow-up period by baseline smoking status (reference category ‘never smoker’ at baseline in 2005). Models adjusted for birth year, monthly income, urban or rural residence in 2005, health insurance coverage, alcohol drinking, physical activities, body mass index, and self-reported health

There was an increasing trend in mortality risk associated with daily cigarette consumption. The hazard ratio of mortality for former heavy smokers compared with never smokers (HR 1.7 [95 % CI 1.2–2.5]) was higher than that of former light smokers (HR 0.9 [95 % CI 0.7–1.2]) and former medium smokers (HR 1.5 [95 % CI 1.0–2.1]). Similarly among current smokers, the hazard ratios of premature mortality compared with never smokers were highest for heavy smokers (HR 2.4 [95 % CI 1.6–3.6]), followed by medium smokers (HR 1.9 [95 % CI 1.4–2.7]) and light smokers (HR 1.6 [95 % CI 1.2–2.2]).

Cause of death analyses showed that current male smokers had a significantly increased risk of death from cardiovascular disease (HR 3.9 [95 % CI:1.8–8.1]) after adjusting for demographic factors, behavioral variables, body mass index, and self-reported overall health. Current smokers also had a higher mortality risk for deaths from injury (HR 1.7 [95 % CI 1.1–2.6]) and from other causes (HR 1.8 [95 % CI 1.1–3.0]) after adjusting for potential covariates. Former smokers had a lower risk of dying from cardiovascular diseases (HR 0.4 [95 % CI 0.2–0.8]) and from injury (HR 0.5 [95 % CI 0.3–0.7]) than current smokers, though former smokers had a moderate increase in risk for dying from cardiovascular deaths compared to those who never smoked (HR 1.6 [95 % CI 0.7–3.4]). For other causes of death, effects of smoking were not significant comparing mortality risk for former smokers and current smokers (HR 0.9 [95 % CI 0.6–1.4]), but we noted that former smokers had a moderately higher risk than never smokers (HR 1.6 [95 % CI 1.0–2.6]). There was no significant increase in risk of cancer mortality among current smokers or former smokers compared with never smokers. We did not conduct analysis on association between the daily consumption of cigarettes and cause-specific mortality risk in the Cox hazard model as there were very few deaths in each category.

The longitudinal smoking status during the 2005–2009 period was associated with subsequent survival between 2009 and 2012 (Table 4). For males, never smokers were more likely to survive from 2009 to 2012. After full adjustment for demographic characteristics, risk behaviors, and physical activity in 2009, current smokers between 2005 and 2009 experienced highest mortality hazards on the next 4 years compared to never smokers (HR 2.1 [95 % CI 1.4–3.4]). The risk of dying reduced if people quit smoking during the 2005–2009 period (HR 1.5 [95 % CI 0.7–3.3]). Risk of mortality was even lower among long–term quitters (HR 1.4 [95 % CI 0.9–2.2]).

**Table 4:** Smoking (2005–09) and subsequent outcomes (2009–12) for males in the Thai Cohort Study: survival and mortality

Longitudinal smoking status (2005–09)	2012 % Survival by smoking status		Hazard ratios (95 % CI) for mortality from 2009 to 2012 by smoking status
	Survived	Died	
	(*n* = 22,026)	(*n* = 161)	
Never smoke	99.5	0.5	Reference
Quit smoking from 2005	99.1	0.9	1.4 [0.9–2.2]
Current smoking in 2005 and quit in 2009	99.1	0.9	1.5 [0.7–3.3]
Current smoking in 2005 and in 2009	98.9	1.1	2.1 [1.4–3.4]

Hazard ratios compare mortality over the follow-up period by longitudinal smoking status over the previous period (reference category ‘never smokers’ in both 2005 and 2009). The model includes age category (five groups), monthly household income (2009), urban or rural residence in 2009, health insurance coverage (2005), alcohol drinking (2009), physical activities (2009), body mass index (2009), and self-reported health (2009)

## Discussion

We report on smoking and mortality from 5- and 7-year follow up of a large cohort of Thai adults. Smoking was almost exclusively a health risk for males because very few Thai females smoke. Among males, more than 90 % of cohort members were born after 1960 (aged ≤45 years in 2005 baseline). Never smokers had a higher proportion of the younger birth cohort (aged ≤25 years in 2005 baseline). Current smokers in the cohort were more likely to die from all-cause mortality than former smokers or non-smokers. The hazard of all-cause mortality compared to non-smokers increased with the daily amount of cigarette consumption among both current and former smokers. Cause of death analysis showed that current male smokers had a significantly higher risk of cardiovascular disease mortality (HR 3.9 [95 % CI 1.8–8.1]). Current male smokers in both 2005 and 2009 experienced highest subsequent all-cause mortality hazards (HR 2.1 [95 % CI 1.4–3.4]). The risk of dying reduced if people quit smoking, and mortality risk was even lower among long-term quitters.

Our findings confirm the smoking and mortality relationship previously reported in Western countries [1, 3]. Our findings also show that mortality risk increased with the daily amount smoked among both former and current smokers in the Thai population, as has been reported in the West [7]. Our study further provides longitudinal nationwide evidence supporting various reports from Thailand, including the important adverse effects of smoking among the Thai population as shown by the Burden of Disease study in 2009 [35]. Our study reveals a strong association between smoking and cardiovascular death in young to middle age Thais, which is consistent with the results of other studies [36]. Our study further confirms that cessation of smoking among Thais can lower mortality from specific causes such as cardiovascular diseases [17–19]. In addition, association between smoking and injury has been noted in other studies [20, 21]. Plausible scenarios include fires or road crashes caused by smoking when falling asleep, distracted, or inattentive, though the demographic and psychological characteristics of smokers may be confounders. Our findings also support a previous study on smoking and mortality among rural Thais, especially among men [37] and provide additional evidence on the benefits of smoking cessation on cause-specific mortality.

Compared to Western countries, female smoking is particularly low in Thailand [34, 38]. This is an enormous advantage, for half the Thai population could avoid exposure to one of the deadliest substances available publicly. In another setting current female smokers, compared to female non-smokers, had double all-cause mortality rates and triple stroke and cerebrovascular deaths [39]. Furthermore, smoking cessation among females reduced all-cause deaths by 25 % and cardiovascular deaths by 60 % [8]. However, trends in female smoking in some middle- and low-income countries are increasing [40]. Hence, countries with low rates among females should actively support such culturally-conferred public health advantage enjoyed by the population.

The main strength of our study is its large participation by Thai adults who responded to a comprehensive baseline questionnaire addressing a wide array of social and health characteristics. This information, combined with longitudinal analyses, allows elimination of many confounders and this helps to establish causal pathways such as the link between smoking and mortality. Our cohort members share certain important characteristics with the general Thai population including similar modest incomes, similar geographic distribution, and similar ethnicity and religion. Cohort members have completed high school education, which could have positive influence on the uptake of health promotion including smoking cessation [41]. A study by Pachanee found the advantage of education in reducing smoking, after adjusting for other factors [42]. Given relatively higher education among cohort members than general Thais and associated lower health risks and better outcomes, our findings confirm the importance of intensifying the campaign on smoking control, with a clear focus of reducing male smoking and preventing female smoking.

We acknowledge some limitations of our study as follows. First, cohort members were not asked about reasons for smoking cessation and some could be due to health reasons. We used self-reported health as a proxy for overall health in our analyses. We also conducted a sensitivity analysis to test similar models for all-cause mortality with Cox regression but excluded cohort members who reported chronic diseases (e.g., cancers, ischemic heart diseases, cerebrovascular diseases, and diabetes) at the 2005 baseline. The following HRs by smoking status (1.3 [95 % CI 1.0–1.6] for former smokers and 1.8 [95 % CI 1.4–2.4] for current smokers) during the 7-year follow up (up to August 2012) from this sensitivity analysis were similar to the corresponding figures (1.2 [95 % CI 0.9–1.5] for former smokers and 1.8 [95 % CI 1.4–2.3] for current smokers) in the Results section. The models in the sensitivity analysis for cause-specific outcomes only excluded people who reported certain diseases at the 2005 baseline which could be related to the specific cause of deaths. For example, for analysis of cancer deaths, cohort members who reported cancers at the 2005 baseline were excluded. For analysis of CVD mortality, we excluded cohort members with ischemic heart disease, cerebrovascular disease, and diabetes from the 2005 baseline. For all other causes of death, we restricted to cohort members who did not report diabetes in 2005. The patterns of HR for smoking status from the sensitivity analysis were similar to those reported in the Results section. However, the HRs for CVD mortality in the sensitivity analysis (2.5 [95 % CI 1.0–6.3] for former smokers and 5.1 [95 % CI 2.1–12.4] for current smokers) were higher than the corresponding figures reported in the Results section (1.6 [95 % CI 0.7–3.4] for former smokers and 3.9 [95 % CI 1.8–8.1] for current smokers).

We also note possible limitation of the study relating to the lack of information on types of tobacco smoked in our study; this could be important if mortality risk depends on tobacco type. However, a recent longitudinal study in rural Thailand noted that there was no statistical difference in mortality risk between hand-rolled and manufactured tobacco [37]. Nevertheless, information on types of tobacco used could be helpful for designing interventions. For instance, hand-rolled tobacco is much cheaper than manufactured cigarettes and if a high proportion of current smokers uses hand-rolled cigarettes, increasing tax for such product may reduce its demands. In addition, information on the history of smoking (e.g.*,* age at initiation or duration, the length of cigarette cessation for quitters) was not available and smoking cessation was reported at baseline and at 4-year follow-up. It is therefore possible that respondents may resume smoking during or after that period. Such misclassification is more likely to underestimate smoking and mortality risks.

We acknowledge that more than 90 % of current male smokers in the cohort were aged less than 45 years (and two-thirds were aged less than 35 years) when they began to be followed up in 2005. Also, the mean age at smoking initiation among male daily smokers was 17.3 years in Thailand [25]. These may explain why we did not observe a significant increase in cancer mortality (including lung cancer). More follow-up will be informative on smoking and cancer.

Smoking rates and patterns of smoking related diseases vary across regions in Thailand. For example, the Southern region (24.6 %) had the highest smoking rate in the country in 2013 followed by Northeast (22.8 %), the North (20.0 %), and Central (18.2 %) zones [43]. However, 53.5 % of the new cases of lung cancer in 2009 were from Bangkok Metropolitan Area, while only 18.0 and 13.0 % were from the Northeast and the South [44]. Regional differentials in association between smoking and subsequent mortality may be analysed further in the Thai Cohort Study as deaths accumulate in the future.

The implementation of tobacco control in Thailand has been recognised as a success and smoking prevalence has already reduced. The current National Tobacco Control Strategy 2012–2014 focuses on prevention of smoking initiation and reduction of current smoking [45]. Our study shows clearly the accelerated mortality associated with smoking at an early stage of the epidemic and among smokers in early middle age. The disease burden from smoking would be expected to increase rapidly among current smokers who continue to smoke and this will get worse if adolescents continue to initiate smoking at progressively earlier ages. The study also helps quantify the expected benefit for Thailand if smoking rates among males fall and smoking rates among females remain very low. Both outcomes will lead to mortality reduction due to prevention of the large set of chronic diseases attributable to smoking. Even over a short period of study including mostly young adults, we were able to detect substantial avoidable smoking related mortality. These local data provide clear evidence not previously available. As expected, the adverse effects of tobacco are very evident in Thailand; most importantly, quitting is beneficial.

## Abbreviations

HR: Hazard ratios
TCS: Thai Cohort Study
CVD: Cardiovascular diseases
CI: Confidence interval
